# Do whole body impedance cardiography estimates of left ventricular structure, volumes and function correlate with the gold standard of cardiac magnetic resonance imaging?

**DOI:** 10.1186/1532-429X-18-S1-P194

**Published:** 2016-01-27

**Authors:** Mariam Narous, Eric Yee, Karen Cowan, Nowell M Fine, Yoko Mikami, James A White, Derek V Exner

**Affiliations:** 1 Libin Cardiovascular Institute of Alberta, Calgary, AB Canada; 2 grid.22072.350000000419367697Cardiac Sciences, University of Calgary, Calgary, AB Canada; 3 grid.22072.350000000419367697Stephenson Cardiac Imaging Centre, Libin Cardiovascular Institute of Alberta, University of Calgary, Calgary, AB Canada

## Background

Cardiac magnetic resonance (CMR) is considered a gold standard for assessing left ventricular (LV) structure, volume and function. Impedance cardiography has been purported to provide similar information, including a surrogate measure of LV ejection fraction (EF). We sought to determine whether impedance cardiography estimates correlate with CMR values.

## Methods

Consecutive patients (n = 204) undergoing a standard clinical CMR using either 1.5T or 3T MRI were enrolled. Whole-body impedance cardiography, using the Non-Invasive Cardiac System (NICaS), was performed within 14 days of the CMR. At least 5 consecutive NICaS measurements, calculated every 20 seconds, were averaged to estimate LV stroke volume (SV), cardiac output (CO) and the surrogate of an LV EF < 55% (i.e., Granov Goor Index < 10). Short axis cine imaging was performed in accordance with CMR Society guidelines. CMR and NICaS results were compared using linear regression. Bland-Altman (BA) plots were incrementally used to evaluate individual variability in modality correlation over the range of data observed. The capacity of NICaS to predict a CMR-based LV EF < 55% was determined via receiver operating characteristic curve (ROC) area under the curve (AUC) analysis.

## Results

**Figure 1:**
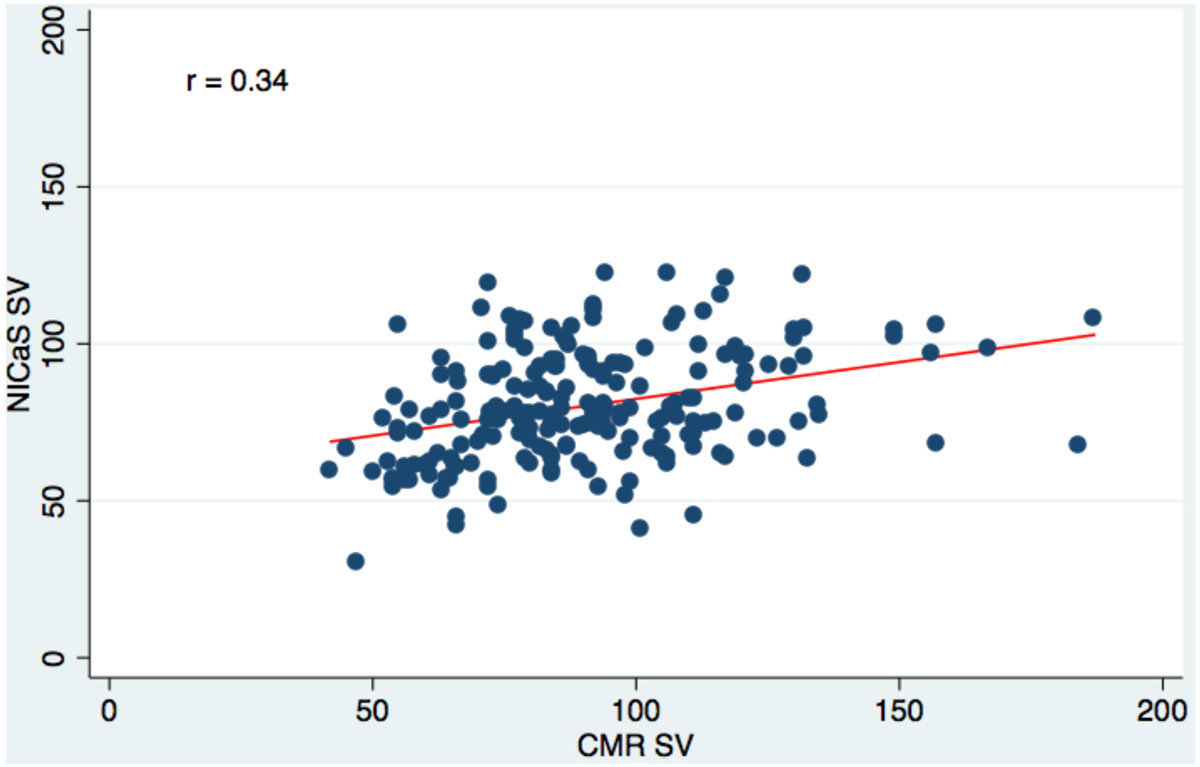
**Scatterplot of SV correlation between NICaS and CMR**.

**Figure 2:**
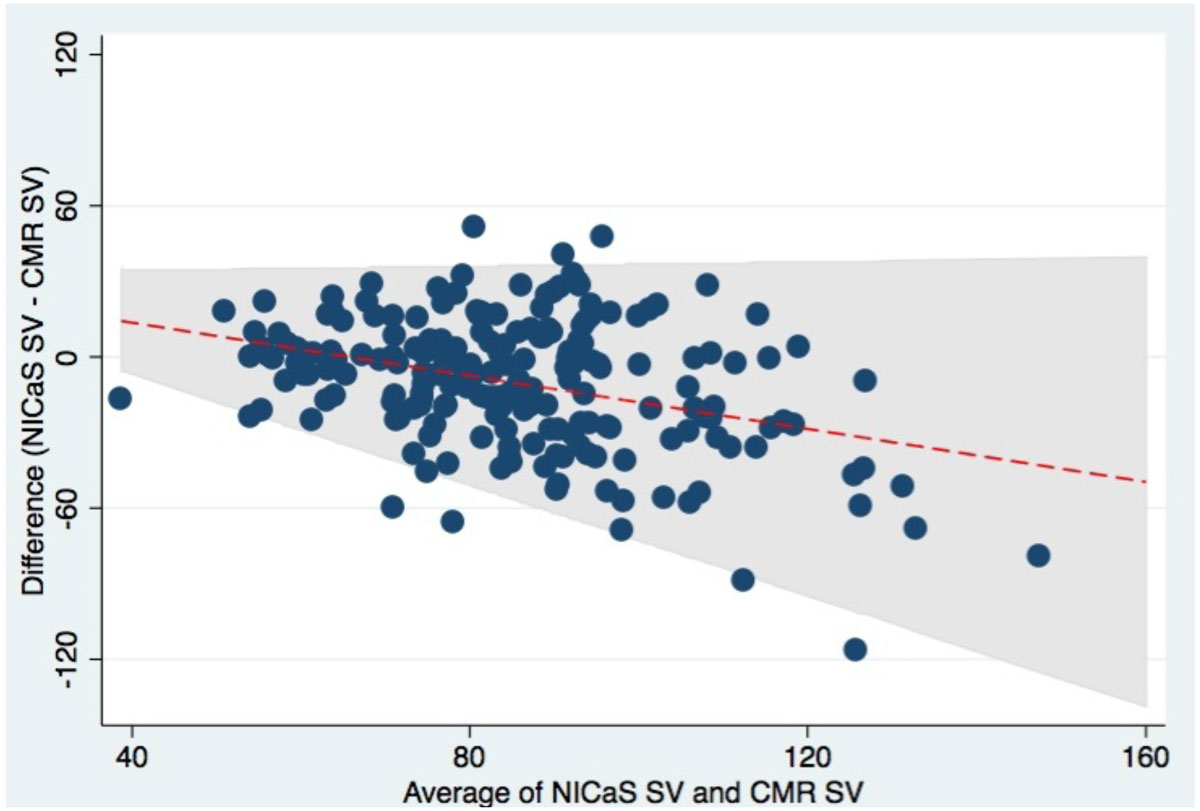
**Bland-Altman plot of individual NICaS SV and CMR SD values**.

The study population included 85 (42%) women, had a mean age of 55 years and a mean CMR EF of 57% (range 22% to 82%). A modest, significant, linear correlation was found between NICaS and CMR LV SV (r = 0.34; p < 0.0001) (Figure 1), though there was substantial variability within subjects over the range of values (Figure 2). Similar results were observed between NICaS and CMR CO (r = 0.19; p = 0.007). No significant linear correlation between the NICaS estimate of LV EF and CMR LV EF was observed (r = 0.13; p = 0.07). There were 72 (35%) subjects with a CMR EF < 55%. ROC analysis showed an AUC for the NICaS LV EF surrogate of 0.53 for predicting a CMR LV EF < 55% with a sensitivity of 41% (95% CI: 30% to 53%) and specificity of 68% (95% CI: 59% to 76%).

## Conclusions

NICaS estimates of LV structure, volume and function were only modestly correlated with CMR values and the capacity of impedance cardiography to predict CMR values was limited (r^2^ for CO = 4% and r^2^ for SV = 11%). Further, significant variability within patients was seen. NICaS was not reliable for identifying patients with a reduced LV EF, as evidenced by a low ROC area under the curve, modest sensitivity and poor specificity. These data do not support the use of NICaS impedance cardiography as a surrogate marker of LV structure, volume and function as compared to the gold standard of CMR.

